# The role of anxiety and trauma in predicting school avoidance among students: a structural equation modeling analysis

**DOI:** 10.3389/fpsyg.2025.1687797

**Published:** 2025-11-17

**Authors:** Haoyu Wang, Qixiu Qin

**Affiliations:** 1 China West Normal University, School of Foreign Languages, Nanchong, China; 2 Liuzhou Polytechnic University, Guangxi, China

**Keywords:** adolescent anxiety, traumatic experiences, school avoidance behavior, emotion regulation, structural equation modeling, mental health intervention, educational psychology

## Abstract

This study investigated how anxiety and traumatic experiences contribute to adolescents’ school avoidance by constructing a dual-path mediation model. In this model, emotion regulation was tested as a mediator linking anxiety to school avoidance, while psychological resilience was examined as a mediator linking traumatic experiences to school avoidance. A total of 500 school students from eastern China participated in the survey. Structural equation modeling (SEM) was used to analyze the direct and interactive effects of the two risk factors, assess two mediating pathways, and investigate potential group differences by gender and grade level. The research employed a combination of methods—including questionnaire surveys, descriptive statistics, correlation analysis, multiple regression, and model fitting—to comprehensively evaluate five key variables: anxiety, trauma, emotion regulation, resilience, and school avoidance. The results showed that both anxiety and traumatic experiences were significant predictors of school avoidance, and their interaction further intensified avoidance behavior. Emotion regulation partially mediated the effect of anxiety on school avoidance, while psychological resilience served as a protective mediator between trauma and avoidance. This study is the first to integrate anxiety and trauma into a single analytical framework, highlighting the complex interactions between risk and protective factors. It also contributes new empirical insights into gender and grade-level variations. These findings underscore the importance of incorporating emotion regulation and resilience-building strategies into school curricula and teacher training programs. This study is the first to place anxiety and trauma within a single analytical framework, underscoring how risk and protective factors interact to shape school avoidance. It further offers empirical evidence of variations by gender and grade level. Taken together, the results highlight the value of incorporating emotion regulation and resilience training into school curricula and teacher preparation, while also providing a solid foundation for developing more precise and sustainable school-based mental health services.

## 1 Introduction

Adolescence is a critical stage for social and psychological development. Schools play a dual role during this period—not only providing academic instruction but also serving as key environments where adolescents form self-identity and build social relationships. In recent years, however, a growing number of adolescents have exhibited school avoidance behaviors, such as frequent absences, skipping classes, and even intense fear or resistance toward attending school. These behaviors not only hinder academic performance but also heighten the risk of psychological issues like self-harm and self-devaluation, posing serious threats to adolescents’ overall development. Previous research has shown that adolescents with high levels of anxiety are more likely to engage in school avoidance, often due to challenges in regulating negative emotions (Zhao et al., 2024). Traumatic experiences—such as family conflict, peer bullying, or significant life events—can increase the risk of emotional disorders and weaken students’ capacity to adjust to school, particularly in areas such as engagement, classroom participation, and attendance (Tang et al., 2020). In this study, school avoidance is understood as one behavioral manifestation of impaired adjustment, and within the Chinese basic-education context, junior secondary students appear especially vulnerable, as academic pressure and interpersonal conflict often intensify these risks. Emotion regulation deficits may worsen these challenges by intensifying internalizing symptoms and undermining long-term mental health (Hatzenbuehler et al., 2008). Therefore, understanding how anxiety and trauma influence school avoidance through the mechanisms of emotion regulation is crucial for developing targeted intervention strategies. Among the psychological risk factors impacting adolescent school adjustment, anxiety and trauma are especially prominent. Anxiety often manifests as persistent worry, tension, and avoidance behaviors, which interfere with students’ ability to engage fully with academic and social environments. Traumatic experiences—ranging from family dysfunction to peer victimization—can have long-lasting effects on adolescents’ emotional regulation systems, reducing their capacity to cope with stress. Evidence suggests that both anxiety and trauma not only compromise mental health directly but also impair behavioral functioning indirectly by weakening emotion regulation abilities (Sawyer et al., 2001).

Emotion regulation functions as a central psychological resource, allowing adolescents to manage negative emotions and cope with stress. The development of emotion regulation is critical for shaping how young people respond to environmental demands and psychological challenges. Structural equation modeling studies have found that adolescents lacking effective emotion regulation strategies are more vulnerable to internalizing problems, including anxiety and depression, in the face of negative emotional experiences (Tortella-Feliu et al., 2010). Moreover, strong links have been identified among family support, positive psychological development, and the use of adaptive regulation strategies, indicating that emotion regulation can buffer the impact of psychological stress and help adolescents maintain emotional balance (Rahimi et al., 2024). These findings support the conceptualization of emotion regulation as a mediating factor between anxiety, trauma, and school avoidance—both theoretically and empirically.

Although research on adolescent school avoidance has advanced in recent years, several important gaps remain. First, most existing studies examine anxiety or trauma as isolated predictors, with few integrating both within a unified analytical framework. For example, some scholars have explored the impact of anxiety on academic engagement from cognitive and behavioral perspectives, highlighting its detrimental effects in specific contexts while neglecting the profound psychological consequences of traumatic experiences (Wangsiriwech et al., 2019). Second, although long-term associations between anxiety, avoidance behaviors, and emotional disorders have been established, few studies have examined the underlying psychological mechanisms—particularly in adolescents—leaving structural modeling approaches underutilized (Jacobson and Newman, 2014).

Early research largely focused on the adverse effects of anxiety or depression on adolescents’ school adjustment, but most remained at a broad correlational level and offered little insight into the underlying mechanisms (Sawyer et al., 2001). As scholarship advanced, a longitudinal study by Hatzenbuehler et al. (2008) showed that deficits in emotion regulation significantly exacerbated internalizing symptoms, suggesting that emotion regulation may be a key process through which risk factors translate into maladaptive outcomes. However, such work often concentrated on single risk variables and was therefore unable to reveal the interplay of multiple stressors. In the 2010s, researchers began to employ structural equation modeling to test the mediating role of emotion regulation between negative emotions and psychological symptoms (Tortella-Feliu et al., 2010). Although this represented methodological progress, most studies still analyzed anxiety or trauma in isolation. By 2017, meta-analyses had confirmed the differential effects of various emotion regulation strategies on adolescent anxiety and depression (Compas et al., 2017; Schäfer et al., 2017), yet they continued to treat anxiety and trauma separately, leaving their combined influence on school-related behaviors unexplored. Parallel work on school attendance and avoidance highlighted the impact of traumatic experiences on reduced classroom engagement and increased absenteeism (Perfect et al., 2016), and Kearney et al. (2019) introduced a multi-tiered systems of support framework to understand school refusal. Nevertheless, these studies did not incorporate anxiety, trauma, and protective factors such as emotion regulation and resilience within the same model. More recent research in China underscores this gap. Fang et al. (2022), for example, found that bullying-related anxiety was closely associated with resilience, with emotion regulation emerging as a key dimension. Taken together, prior research has progressed from correlational studies to pathway modeling and intervention work, yet it still lacks an integrated framework that combines multiple risks and protective factors. In particular, little is known about how anxiety and trauma jointly operate and interact with emotion regulation and resilience to shape school avoidance. To address these gaps, the present study proposes a dual-path structural equation model to provide a more comprehensive understanding of the mechanisms underlying adolescents’ school avoidance.

Structural modeling studies have shown that anxiety, depression, and hopelessness frequently co-occur and jointly predict difficulties in school adjustment (Cunningham et al., 2008), while emotional dysregulation and avoidant coping are key mechanisms underlying the anxiety–avoidance link (Briere et al., 2010). Without early intervention, these patterns may solidify into chronic maladaptive behaviors (Paulus and Siep, 2021). This study makes several contributions to the literature. First, it constructs and validates a dual-path mediation model in which anxiety and trauma influence school avoidance through emotion regulation, clarifying both their interaction and underlying mechanisms. Second, drawing on findings by Cherewick et al. (2024) regarding the buffering role of social support, the study investigates how emotion regulation training affects different gender and grade groups, offering evidence for targeted intervention strategies. Third, informed by Pedrini et al. (2022)‘s systematic evaluation of school-based emotion regulation programs, the study proposes practical recommendations aimed at narrowing the gap between research and application. Taken together, these contributions enhance the theoretical foundation of adolescent psychological adjustment and offer scientific guidance for implementing emotion regulation interventions in school mental health services and education policy.

## 2 Review

School avoidance is typically characterized by chronic absenteeism, refusal to attend classes, and intense fear or distress associated with the school environment. Unlike truancy—which is often linked to antisocial behavior or a lack of interest in learning—school avoidance is more deeply rooted in underlying emotional disturbances or psychological disorders (Place et al., 2000). Scholars commonly distinguish between two forms of school avoidance: proactive, driven primarily by anxiety or fear of negative evaluation, and passive, associated with apathy, low academic motivation, or insufficient support from family and school (Elliott and Place, 2012). Empirical evidence supports the validity of this distinction. For instance, Delgado et al. (2019), using latent class analysis, identified one subtype of school avoidance primarily motivated by negative emotions and fear of evaluation, and another more strongly related to the pursuit of external attention or a lack of environmental support. More recently, Chen (2024) reported that adolescents experiencing heightened anxiety and whose parents exhibited poor psychological health were more likely to show avoidance behaviors, while those perceiving inadequate family support tended to display more passive forms of withdrawal. Nevertheless, commonly used assessment tools—such as questionnaires, self-reports, and teacher observations—although useful for classification, remain insufficient to capture the complex psychological origins and developmental trajectories of these behaviors (Thambirajah et al., 2008). This underscores the need for mechanism-oriented, theory-driven models that integrate risk factors such as anxiety and trauma with protective factors such as emotion regulation and resilience, thereby providing a more comprehensive framework for understanding school avoidance among adolescents.

A growing body of research highlights the long-term psychological impact of early traumatic experiences—such as domestic violence, neglect, and school bullying—on adolescents’ emotional functioning and sense of safety. These experiences can gradually erode feelings of trust and belonging in the school context, thereby fostering avoidance behaviors (Noam and Hermann, 2002). Trauma may also indirectly impair emotion regulation, especially in adolescents with high anxiety sensitivity or neuroticism (Bolouk et al., 2024). However, many trauma-related studies remain descriptive and lack systematic validation of key mediating mechanisms. For example, while anger expression has been linked to both externalizing and internalizing symptoms (Oh and Lee, 2014), the specific regulatory strategies involved in these processes are often underexplored. Although Doba et al. (2022) identified impaired mentalization and regulation as possible mediators between trauma and maladjustment, their model lacked clarity and failed to fully capture inter-variable dynamics. Similarly, Musso et al. (2024) documented the exacerbating effects of trauma-related stress during the COVID-19 pandemic but did not examine variation in emotion regulation strategies or individual differences in regulatory capacity.

Beyond trauma and emotional stressors, contextual factors such as family attachment patterns also play a significant role in school avoidance. Dysfunctional parent–child relationships can trigger anxiety and depressive responses, limit adolescents’ ability to navigate school-based social dynamics, and even increase susceptibility to peer bullying (Lee and Shin, 2021). Longitudinal evidence suggests that emotion regulation deficits serve as a key link between early trauma and later psychological problems, predicting anxiety, depression, and behavioral disturbances (McLaughlin et al., 2011). Adolescents from resource-scarce environments may be especially vulnerable, as limited emotional resources increase the likelihood of maladaptive coping and school disengagement (Ward-Smith et al., 2024). Despite these insights, many studies fail to model how specific regulation strategies mediate the progression from emotional risk to school avoidance.

In recent years, emotion regulation has gained increasing attention in both educational and developmental psychology as a key mechanism linking risk factors to behavioral outcomes. The specific strategies adolescents use to manage negative emotions—such as cognitive reappraisal versus suppression—significantly influence their behavioral responses. Adaptive strategies can buffer against anxiety, while maladaptive ones often intensify psychological vulnerability (Liang et al., 2021; Putwain et al., 2021). This is particularly evident in contexts like test anxiety, where poor regulation often leads to helplessness, motivational decline, and behavioral withdrawal. Moreover, deficits in emotional regulation—especially when interacting with trauma-related stress—predict a range of maladaptive behaviors, including self-harm and social withdrawal (Andersson et al., 2022).

Despite growing empirical support, most existing models fail to clearly specify how individual regulation strategies function as mediators between emotional risk and maladaptive behavior. The conceptual roles of variables often remain vague, and the mechanisms inadequately mapped. For instance, Doba et al. (2022) proposed emotion regulation and mentalization as mediators between childhood trauma and adolescent outcomes but did not distinguish the effects of different strategies or explore complex model pathways. Similarly, Sharma et al. (2024) incorporated emotion regulation as a mediator between trauma and psychopathology but offered only a surface-level explanation. Although Forsyth (2016) applied longitudinal modeling to examine emotional responses and mental health outcomes, dynamic regulatory changes were not fully captured. Zhao and Zhao (2015) explored school connectedness as a mediator in the relationship between emotion regulation and depression but did not clearly define the types or functions of regulation strategies, limiting theoretical depth.

Recent research has further emphasized the role of the family environment in shaping emotion regulation self-efficacy and adolescent adjustment. Secure attachment has been shown to foster adaptive regulation, while insecure relationships correlate with maladaptive strategies like avoidance and suppression, contributing to school avoidance behavior (Pan et al., 2021). Trauma exposure may also lead to behavioral withdrawal when regulation systems are underdeveloped. For example, Tierens et al. (2012) found that adolescents who experienced traumatic events, such as accidents, were more likely to develop persistent avoidance tendencies if their regulation capacities were impaired. Panwar et al. (2023) further reported that the impact of impulsivity and anxiety on school functioning is largely mediated by differences in emotional regulation abilities. Similarly, Shahnazdoust et al. (2022) demonstrated that maladaptive emotion regulation strategies mediate the relationship between emotional abuse and self-harming behaviors.

Although these findings underscore the pivotal role of emotion regulation, they also reveal significant gaps in integrated modeling. In real-world scenarios, anxiety and trauma often co-occur, influencing adolescents’ emotional and behavioral functioning in complex ways. Focusing on isolated variables obscures the interplay between multiple risk factors. Importantly, emotion regulation may serve distinct mediating roles across different pathways, yet this dual-pathway function remains underexplored. To address these limitations, the present study proposes a structural equation model that positions emotion regulation as the central mediator linking anxiety and trauma—conceptualized as dual risk factors—to school avoidance. This model aims to systematically examine how these variables interact and to clarify the regulatory processes involved. By doing so, it bridges theoretical gaps and offers structured, mechanism-based guidance for designing effective interventions in school mental health services.

## 3 Empirical methods

### 3.1 Research objectives and hypotheses

This study draws on empirical data from 500 middle school students in eastern China, aiming to construct and test two mediating pathways and their interaction model: one pathway examines how anxiety (ANX) influences school avoidance (EBSA) through emotion regulation (ER), while the other explores how traumatic experience (TRAUMA) affects school avoidance through psychological resilience (RES). The ultimate goal of this study is to comprehensively reveal the psychological mechanisms underlying adolescent school avoidance behavior, thereby providing precise theoretical support for educational and psychological interventions.

#### 3.1.1 Research objectives

(1) To quantify the direct effects of anxiety and trauma on school avoidance behavior: Using structural equation modeling (SEM), this study estimates the marginal effects of anxiety levels and traumatic experiences on school avoidance, clarifying their relative contributions to avoidance tendencies.
(2) To examine the mediating mechanisms of emotion regulation and psychological resilience: Specifically, it investigates whether emotion regulation (ER) serves as a partial mediator in the pathway from anxiety to avoidance, and whether resilience (RES) transmits the effect from trauma to avoidance—thus revealing how risk and protection factors jointly shape avoidance behavior.
(3) To assess the interactive amplification effect of anxiety and trauma: After controlling for mediators and covariates (gender, grade), the interaction term (ANX × TRAUMA) is tested to evaluate the joint effect of anxiety and trauma, and to assess whether the “cumulative risk model” provides enhanced explanatory power for school avoidance.

#### 3.1.2 Research hypotheses

*H1*: Anxiety levels significantly and positively predict school avoidance behavior.

*H2*: Traumatic experiences significantly and positively predict school avoidance behavior.

*H3*: Emotion regulation partially mediates the relationship between anxiety and school avoidance.

*H4*: Psychological resilience partially mediates the relationship between traumatic experience and school avoidance.

*H5*: The interaction between anxiety and traumatic experience significantly and positively amplifies school avoidance behavior.

#### 3.1.3 Theoretical SEM model

Latent Variable 1: Anxiety (ANX) — including social anxiety, test anxiety, and generalized anxiety.

Latent Variable 2: Traumatic Experience (TRAUMA) — including domestic violence, school bullying, and major loss events.

Latent Variable 3: Emotion Regulation (ER) — including emotional awareness, emotion control, and self-regulation.

Latent Variable 4: Psychological Resilience (RES) — including resilience, problem-solving coping, and positive cognition.

Latent Variable 5: Emotional-Based School Avoidance (EBSA) — including frequent leave requests, willingness to skip class, and intense fear of being at school.

#### 3.1.4 Model paths

See Figure 1.

**Figure 1:**
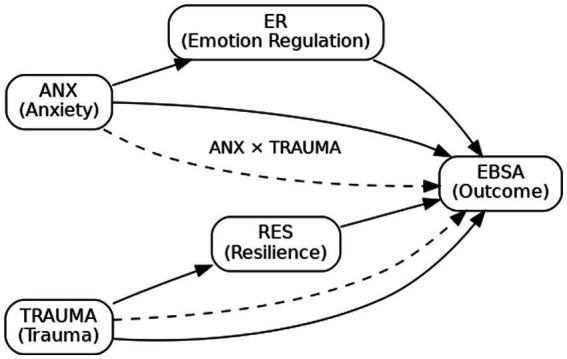
Hypothesized structural equation model (SEM) path diagram.

##### 3.1.4.1 Participants and sampling

This study targeted in school adolescents aged 12–17 in eastern China (covering both junior and senior high school stages). The sample size was pre-set at 500 to meet the statistical power requirements of structural equation modeling and Bootstrap mediation testing. A stratified random sampling method was adopted, stratified by gender, grade level, and school type to ensure good sample representativeness. Inclusion criteria: (1) aged between 12 and 17 years; (2) currently enrolled in school and without severe cognitive impairments; (3) informed consent obtained from both the participant and their legal guardian. Exclusion criterion: currently undergoing pharmacological treatment for psychiatric disorders. Through this design, the data collected in this study demonstrate high external and internal validity, providing a solid foundation for subsequent SEM analysis.

#### 3.1.5 Instruments

See Table 1.

**Table 1:** Instruments and reliability of latent variables.

Latent variable	Items used	Dimensions/Subdimensions	Cronbach’s *α*	Notes
Anxiety (ANX)	Q1–Q5	Cognitive anxiety, emotional response, physiological symptoms	0.89	Derived from 5 Likert-scale items assessing exam/social/general anxiety
Trauma exposure (TRAUMA)	Q6–Q9	Diversity of traumatic events, subjective trauma intensity	0.86	4 items covering domestic violence, bullying, major loss, fear/helplessness
Emotion regulation (ER)	Q10–Q13	Emotional awareness, clarity, impulse inhibition, regulation strategies	0.78	4 items; lower reliability, caution needed for statistical interpretation
Resilience (RES)	Q14–Q17	Endurance, positive attribution, goal orientation, social support	0.80	4 items measuring coping and recovery capacity
School avoidance (EBSA)	Q18–Q22	Academic avoidance, spatial avoidance, psychological absence	0.85	5 items capturing observable and latent avoidance patterns

##### 3.1.5.1 Data collection and ethical review

A combination of online and offline methods was used for centralized data collection within schools: the online portion involved distributing questionnaire links via a trusted educational platform, while the offline portion was organized by the research team in classrooms to ensure standardization and completeness of the data collection process. Prior to the start of the study, the research protocol was submitted to and approved by the institutional ethics committee. All participants and their legal guardians were fully informed about the study’s purpose, procedures, and potential risks, and written informed consent was obtained. To protect student privacy, all questionnaires were anonymized, with each participant identified only by a unique code. All data were used solely for academic research and stored on secure, access-restricted servers.

#### 3.1.6 Innovation and academic contribution

This study integrates the dual-pathway perspective of anxiety and trauma, and for the first time systematically quantifies their structural impact on school avoidance behavior.

By introducing two mediating variables—Emotion Regulation (ER) and Resilience (RES)—it addresses the current gap in EBSA research regarding underlying psychological mechanisms.

The use of a Structural Equation Model (SEM) allows for the clear identification of path strengths between variables, contributing to the development of more precise intervention strategies.

### 3.2 Variable explanation

#### 3.2.1 Dependent variable

In this study, school avoidance behavior (EBSA) was defined as a set of strategies used by adolescents in academic and school contexts in response to internal distress or external adverse events, comprising three dimensions: academic avoidance (skipping class, arriving late, leaving early), spatial avoidance (withdrawing from specific school spaces), and psychological absence (being physically present but mentally disengaged due to anxiety or trauma). These dimensions combine observable behaviors with latent psychological states, allowing EBSA to capture the multifaceted impact of anxiety and trauma on school functioning. The construct was measured by averaging the scores of items Q18–Q22 on a five-point Likert scale, so that the resulting index remained on the same 1–5 metric as the individual items. This approach facilitates comparability across participants and makes interpretation straightforward: higher mean scores reflect stronger avoidance tendencies across the three dimensions, whereas lower scores indicate weaker avoidance and greater school engagement. The internal consistency was acceptable (Cronbach’s *α* = 0.85), supporting the reliability of the measure in this study (Table 2).

**Table 2:** Dimensions, items, and indicator meanings of school avoidance behavior (EBSA).

Subdimension	Corresponding items	Indicator meaning
Academic avoidance	Q18, Q19	Reducing engagement in academic tasks through behaviors such as absenteeism, tardiness, or early departure
Spatial avoidance	Q20	Avoiding classrooms or shared campus spaces to escape learning or peer interactions
Psychological absence	Q21, Q22	Physically present in school but mentally disengaged due to anxiety or trauma, showing inattentiveness or social withdrawal

#### 3.2.2 Independent variable

(1) Anxiety level

This scale consists of five items (Q1–Q5) covering three key dimensions: cognitive anxiety (e.g., worry about failure), emotional response (e.g., nervousness), and physiological symptoms (e.g., sweating, trembling). Responses are rated on a 1–5 Likert scale. A Cronbach’s *α* = 0.89 indicates high internal consistency, showing the scale reliably captures adolescents’ tension and unease in high-pressure contexts like exams and social situations. The scale balances subjective feelings and objective manifestations, allowing researchers to comprehensively assess students’ emotional burden levels and providing a solid foundation for analyzing risk pathways in the subsequent model (Table 3).

(2) Trauma exposure

**Table 3:** Operational definition and measurement of anxiety level (ANX).

Item	Content
Theoretical definition	Excessive worry and tension experienced by adolescents in social, exam, and generalized situations
Measurement dimensions	Cognitive anxiety, emotional response, physiological symptoms
Measurement tool	Average score of items Q1–Q5, Likert scale 1–5
Reliability indicator (Cronbach’s α)	0.89

The trauma scale consists of four items (Q6–Q9) that assess both external event types (e.g., domestic violence, bullying) and subjective trauma intensity (e.g., fear, helplessness). A reliability of *α* = 0.86 reflects a balanced coverage of both objective exposure and internal experience, ensuring both depth and breadth in measurement. This scale captures the diversity of trauma experiences and is useful in later analysis of the interaction between trauma, anxiety, and avoidance behaviors, offering a reliable tool for understanding coping under compound adversity.

(3) Emotion regulation

The ER scale uses four core items (Q10–Q13) to assess process-level emotional competencies such as awareness, clarity, impulse control, and use of strategies. While brief, the scale shows basic reliability (*α* = 0.78) and effectively captures key emotion management skills among students. Due to generally low and narrowly distributed scores in the sample, caution is warranted regarding statistical power when evaluating its protective effect. Researchers are advised to consider more detailed multidimensional scales or behavioral logs in future studies to enhance validity.

(4) Resilience

The RES scale is composed of four items (Q14–Q17) targeting resilience skills such as adversity endurance, positive attribution, goal orientation, and social support use. A reliability score of *α* = 0.80 indicates good internal consistency, reflecting students’ capacity to apply positive coping strategies when facing challenges. Given the low and narrowly distributed resilience levels in the sample, the effect may appear only “marginally significant” in statistical analysis, highlighting the need for schools and families to systematically promote resilience training for long-term psychological growth.

#### 3.2.3 Structural relationships between variables

**Figure 2:**
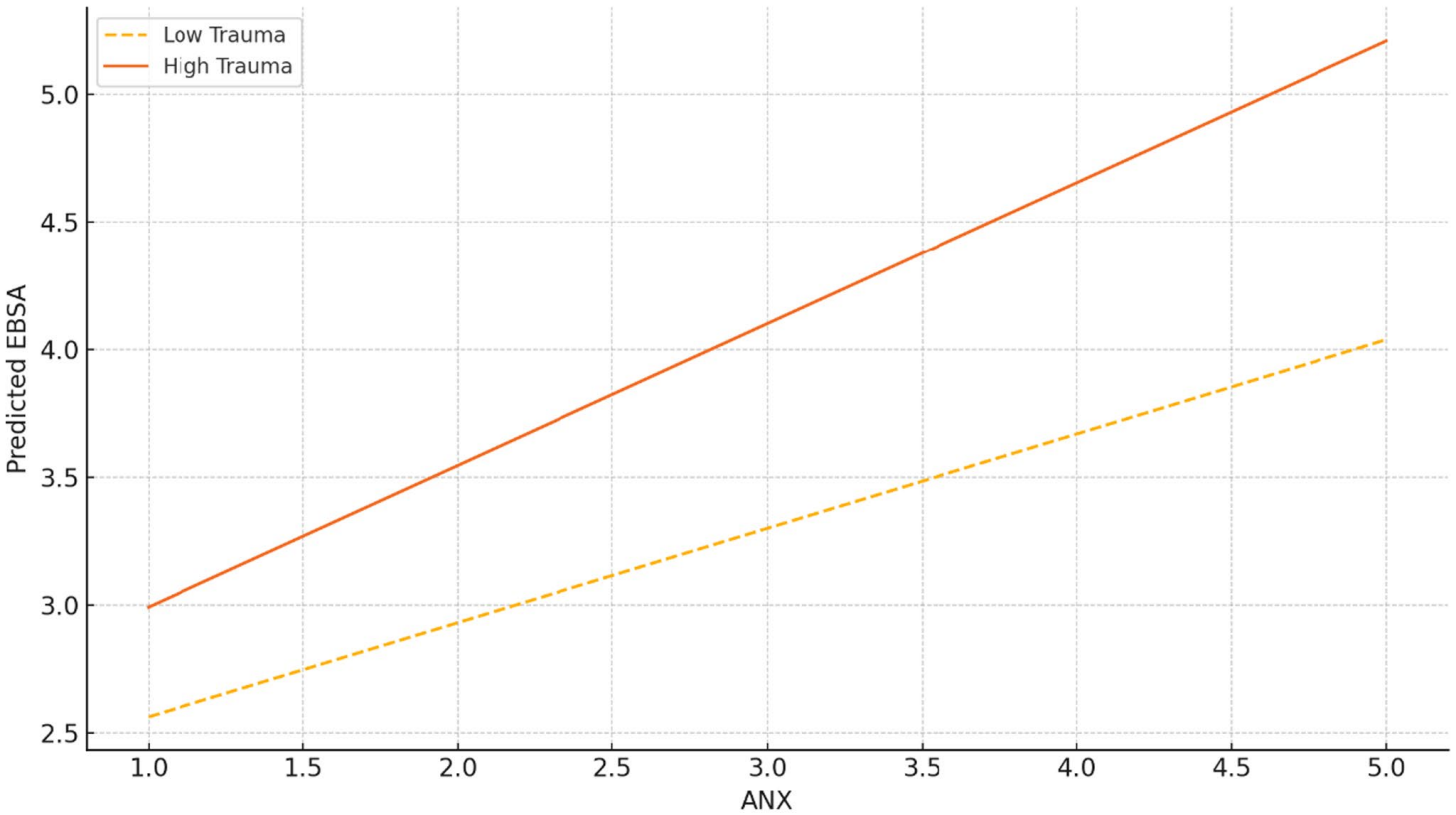
Interaction effect: EBSA vs. ANX by trauma level.

Based on the previously described mathematical model, as well as results from descriptive and correlational analyses, this study constructed the structural model shown in Figure 2, to examine how four exogenous variables—anxiety (ANX), trauma exposure (TRAUMA), emotion regulation (ER), and resilience (RES)—influence school avoidance behavior (EBSA) through direct, interaction, and mediation effects. The model includes:

(1) Main effect paths


$$\text{EBSA}=\alpha +\beta_{1}\mathrm{ANX}+\beta_{2}\text{TRAUMA}+\beta_{3}\mathrm{ER}+\beta_{4}\mathrm{RES}+\varepsilon$$

(2) Interaction effect


$$\begin{array}{l} \text{EBSA}=\alpha +\beta_{1}\;{\mathrm{ANX}}_{c}+\beta_{2}\;{\text{TRAUMA}}_{c}+\beta_{5}\;(\begin{array}{l} {\mathrm{ANX}}_{c}\times \\ {\text{TRAUMA}}_{c} \end{array})+\\ \beta_{3}\;\mathrm{ER}+\beta_{4}\;\mathrm{RES}+\varepsilon \end{array}$$

(3) Mediated sub-models


$$\left\{ \begin{array}{rr} \mathrm{ER} & =\gamma_{1}\;\mathrm{ANX}+\zeta_{1},\\ \mathrm{RES} & =\gamma_{2}\;\text{TRAUMA}+\zeta_{2},\\ \text{EBSA} & =\alpha \prime \prime +\beta \prime_{3}\;\mathrm{ER}+\beta \prime_{4}\;\mathrm{RES}+\varepsilon \prime \prime \end{array}\right.$$

Prior to formally specifying the model, Pearson correlation analysis was conducted for the core variables (see Table 4) to confirm the direction and significance of their relationships, providing empirical support for the path structure. Subsequently, the interaction term between anxiety and trauma was incorporated into both multiple regression and structural equation models to test whether trauma severity amplifies the effect of anxiety on school avoidance. Finally, the Sobel method was employed to evaluate the mediating roles of emotion regulation and resilience in the relationships among anxiety, trauma, and school avoidance. These analyses demonstrated not only the direct promoting effects of anxiety and trauma on avoidance behavior, but also the buffering role of students’ emotion regulation and resilience. The interaction analysis showed that the greater the trauma exposure, the stronger the influence of anxiety on avoidance. The mediation tests further indicated that although ER and RES could mitigate these effects to some extent, their protective functions were limited due to low overall levels in the sample. These findings provide practical implications for designing targeted emotional interventions and support systems for students experiencing high anxiety and trauma (Tables 5, 6).

**Table 4:** Operational definition and measurement of trauma exposure (TRAUMA).

Item	Content
Theoretical definition	Lasting psychological impacts on adolescents resulting from events such as domestic violence, bullying, or major losses
Measurement dimensions	Diversity of traumatic events (e.g., physical harm, emotional neglect); subjective trauma intensity (e.g., fear, helplessness)
Measurement tool	Average score of items Q6–Q9, Likert scale 1–5
Reliability indicator (Cronbach’s α)	0.86

**Table 5:** Operational definition and measurement of emotion regulation (ER).

Item	Content
Theoretical definition	The individual’s ability to identify, understand, and manage emotional responses, including emotional awareness, impulse control, and strategy use
Measurement dimensions	Emotional awareness, emotional clarity, impulse inhibition, goal-oriented regulation, strategy effectiveness
Measurement tool	Average score of items Q10–Q13, Likert scale 1–5
Reliability indicator (Cronbach’s α)	0.78

**Table 6:** Operational definition and measurement of resilience (RES).

Item	Content
Theoretical definition	The ability to respond positively and recover quickly in the face of stress or setbacks, including problem-solving and positive cognition
Measurement dimensions	Endurance under adversity, positive attribution, goal orientation, utilization of social support
Measurement tool	Average score of items Q14–Q17, Likert scale 1–5
Reliability indicator (Cronbach’s *α*)	0.80

## 4 Empirical results

### 4.1 Descriptive statistics of core variables

As shown in Table 7, the sample of 500 adolescents exhibited moderately high levels of anxiety (ANX) and traumatic experience (TRAUMA), with mean values of 3.46 and 3.29, respectively. Both variables also showed relatively large standard deviations (above 1.00), suggesting considerable variability in emotional burden among students, likely due to widespread academic pressure, family conflict, and exposure to cyberbullying. In contrast, students’ scores for emotion regulation (ER) and psychological resilience (RES) were relatively low (means of 2.80 and 2.76, respectively), with smaller variances. This indicates that most students lacked sufficient capacity for emotional adjustment and recovery when faced with stress. The mean score for emotional-based school avoidance (EBSA) was 2.95, close to the midpoint of the scale, with a standard deviation of 1.04. This implies that a significant portion of students tend to adopt avoidance strategies when dealing with academic or interpersonal challenges. This “high-risk–low-protection” pattern suggests that when anxiety and trauma accumulate beyond a certain threshold, and protective resources such as ER and RES are inadequate, school avoidance emerges as a common coping response. Notably, the high standard deviations for anxiety and avoidance indicate the presence of “hidden high-risk” subgroups who may frequently be absent or disengaged from school due to extreme psychological distress. In summary, the descriptive statistics in Table 4 highlight the prevalence of psychological pressure and the inadequacy of coping resources among adolescents. These findings provide important empirical support for subsequent modeling and intervention design. Researchers should pay close attention to risk stratification among students, while practitioners are encouraged to strengthen the development of emotion regulation and resilience skills to reduce the academic and social harm caused by school avoidance behavior.

**Table 7:** Descriptive statistics of core variables.

Variable	Mean	Std. Dev.	Min	Median	Max
Anxiety (ANX)	3.46	1.25	1	4.00	5.00
Traumatic experience (TRAUMA)	3.29	1.22	1	3.00	5.00
Emotion regulation (ER)	2.80	0.78	1	3.00	5.00
Resilience (RES)	2.76	0.67	1	2.50	4.50
School avoidance (EBSA)	2.95	1.04	1.2	3.00	4.80

### 4.2 Pearson correlation matrix

In Table 8, the correlation coefficient between anxiety and school avoidance reached as high as 0.72, while the correlation between traumatic experiences and avoidance was 0.46. Emotion regulation and psychological resilience were negatively correlated with school avoidance, at −0.42 and −0.71, respectively. This correlation pattern reflects a common reality: many students simultaneously face academic pressure, family conflict, and personal trauma. These stressors, when combined, significantly increase the likelihood of avoidance tendencies. At the same time, schools and families often invest insufficiently in cultivating students’ emotional regulation and resilience, meaning that protective resources are inadequate to offset escalating psychological risks. The consequences of this imbalance are severe. On the one hand, high levels of anxiety and trauma make avoidance a common coping strategy, which further undermines students’ interactions with peers and teachers. On the other hand, the lack of effective emotional regulation and resilience support makes it difficult to break avoidance cycles, leading to a self-reinforcing negative pattern. To address this issue, schools must implement systems to identify high-risk students in a timely manner, conduct training in emotion recognition and resilience, and integrate avoidance behavior into routine mental health assessments. These steps are essential to fundamentally reduce adjustment difficulties and promote healthier coping in school settings.

**Table 8:** Pearson correlation matrix.

	ANX	TRAUMA	ER	RES	EBSA
ANX	1.000***				
TRAUMA	0.034	1.000***			
ER	−0.716***	0.367***	1.000***		
RES	−0.567***	−0.522***	0.485***	1.000***	
EBSA	0.720***	0.458***	−0.416***	−0.706***	1.000***

*p* < 0.05*; *p* < 0.01**; *p* < 0.001***.

### 4.3 Path regression analysis (OLS multiple regression)

**Figure 3:**
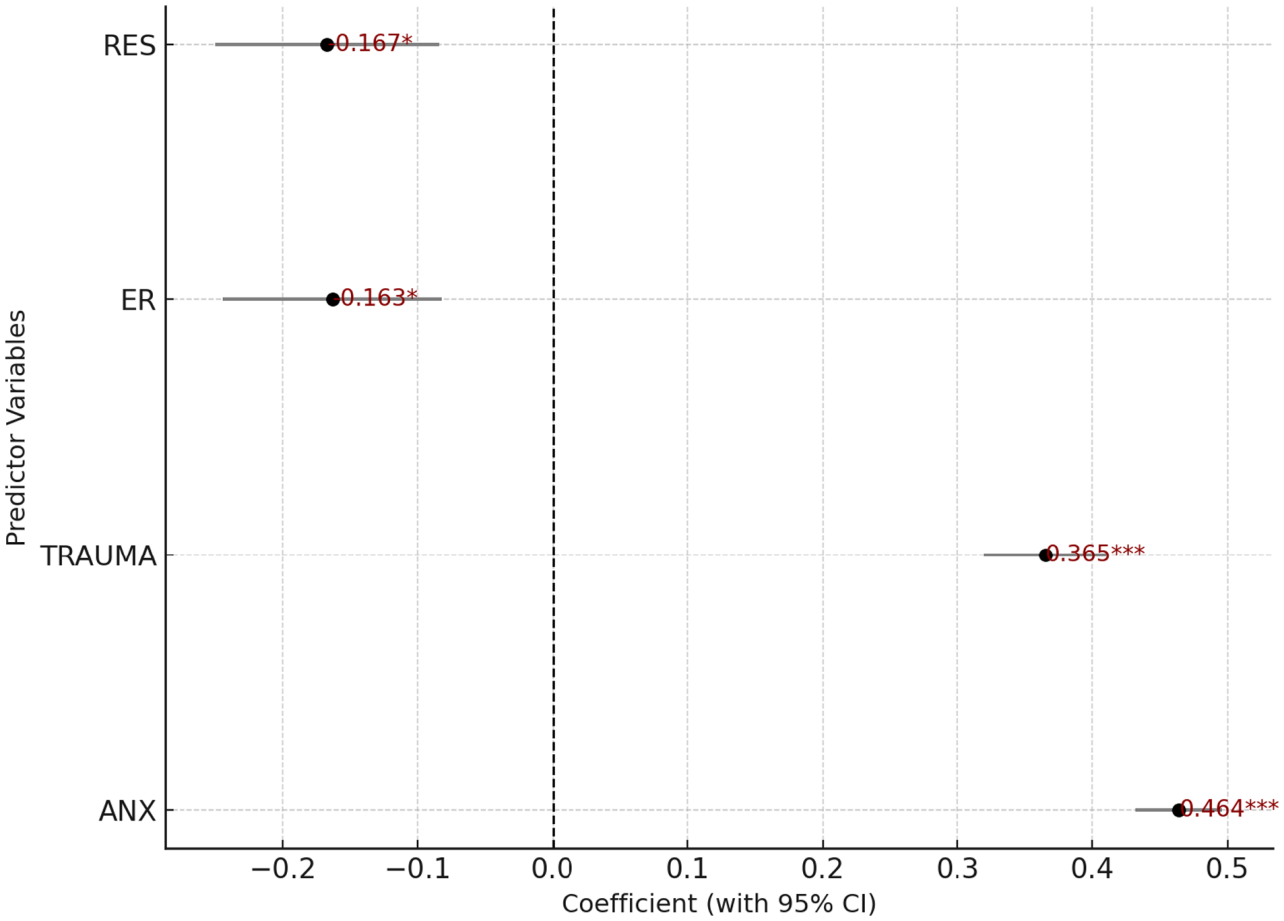
Visualization of regression coefficients with 95% confidence intervals.

In Table 9, the multiple regression results reveal the relative contribution of four predictors to emotional-based school avoidance (EBSA): the regression coefficient for anxiety (ANX) was 0.464 (*p* < 0.001), for traumatic experiences (TRAUMA) 0.365 (*p* < 0.001), for emotion regulation ability (ER) –0.163 (*p* = 0.0438), and for psychological resilience (RES) –0.167 (*p* = 0.0443). Figure 3, a bar chart, visually depicts this pattern: the red bars representing ANX and TRAUMA are significantly taller than the blue bars representing ER and RES, highlighting the dominant role of risk factors in driving school avoidance behavior. While the protective factors operate in the expected negative direction, their effect sizes are limited and only marginally significant. The significance levels of ER and RES lie just above the 0.05 threshold, reflecting a deeper practical dilemma. First, as indicated in the descriptive statistics, students’ overall emotion regulation and resilience capacities are relatively weak and narrowly distributed, limiting the statistical power of these variables to exert strong suppressive effects at the group level. Second, interventions targeting emotion and resilience in schools and families are often fragmented and short-term, lacking the systematic continuity necessary for such skills to accumulate sufficiently to buffer the behavioral impacts of anxiety and trauma. This “risk-heavy, protection-light” reality suggests that reducing school avoidance cannot rely solely on addressing risk factors. Instead, emotion regulation and resilience training must be elevated from peripheral interventions to core, systematic practices. Emotional skill development should be embedded in routine classroom instruction, and these competencies should be given sufficient weight in academic evaluation systems, teacher training, and school-based resource allocation. Only by doing so can the suppressive power of ER and RES move from “marginally significant” to “centrally effective,” ultimately weakening the behavioral grip of anxiety and trauma on students.

**Table 9:** OLS regression coefficients.

Variable	Coefficient (Coef.)	Std. Error	*t*-value	*p*-value
Constant	1.060	0.281	3.772	0.00018
ANX	0.464	0.032	14.642	< 0.001
TRAUMA	0.365	0.045	8.153	< 0.001
ER	−0.163	0.081	−2.021	0.0438
RES	−0.167	0.083	−2.016	0.0443

### 4.4 ANX × trauma → EBSA

In Table 10, the regression coefficient for the interaction term (Anxiety × Trauma) was 0.076 (Standard Error = 0.016, *p* < 0.001), indicating that the impact of anxiety on school avoidance becomes more pronounced as levels of trauma increase. Figure 2 illustrates this interaction effect with two regression lines: among participants with high levels of trauma, the slope between anxiety and school avoidance is steeper; for those with low trauma, the slope is comparatively flatter. This finding suggests that when adolescents experience both high anxiety and significant trauma, their available emotional regulation and psychological support resources may already be depleted, making them more likely to avoid school in search of temporary psychological safety. In real-world practice, addressing anxiety or trauma in isolation is unlikely to break the cycle of avoidance. For the “high anxiety and high trauma” subgroup, interventions must combine trauma recovery with emotion regulation training to effectively reduce school avoidance behaviors stemming from compounded psychological stress.

**Table 10:** Interaction regression coefficients.

Predictor	Coefficient (Coef.)	Std. Error	*t*-value	*p*-value
ANX_c	0.462	0.031	14.871	<0.001
TRAUMA_c	0.363	0.044	8.261	<0.001
ANX × TRAUMA (Inter)	0.076	0.016	4.640	<0.001
ER	−0.168	0.079	−2.126	0.0338
RES	−0.180	0.081	−2.222	0.0268

### 4.5 Path analysis with SEM

**Figure 4:**
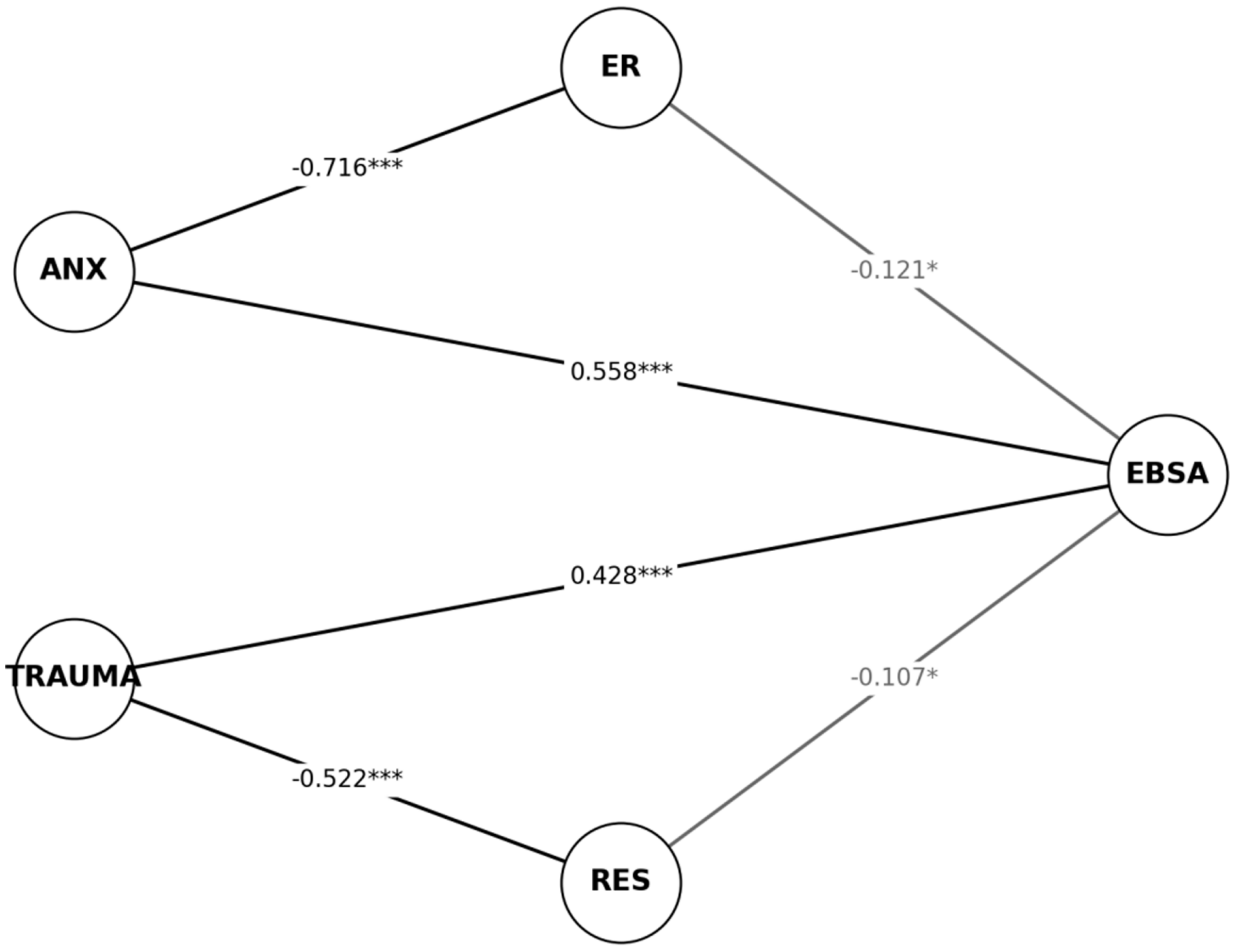
SEM path estimates.

As shown in Figure 4 and Table 11, the path coefficients for anxiety (ANX → EBSA, *β* = 0.558, *p* < 0.001) and trauma (TRAUMA → EBSA, β = 0.428, p < 0.001) were relatively high, confirming that these two risk factors are the strongest drivers of school avoidance behavior. In contrast, emotion regulation (ER → EBSA, *β* = −0.121, *p* = 0.0438) and resilience (RES → EBSA, *β* = −0.107, *p* = 0.0443) showed significant but weaker protective effects. In the path diagram, these links were depicted with thin dashed lines, reflecting the dominance of risk factors over protective mechanisms.

**Table 11:** Path coefficients from SEM analysis.

Path	Standardized coefficient (β)	Std. Error	*t*-value	*p*-value
ANX → ER	−0.716***	0.031	−22.862	< 0.001
TRAUMA → RES	−0.522***	0.038	−13.674	< 0.001
ANX → EBSA	0.558***	0.038	14.642	< 0.001
TRAUMA → EBSA	0.428***	0.052	8.153	< 0.001
ER → EBSA	−0.121*	0.060	−2.021	0.0438
RES → EBSA	−0.107*	0.053	−2.016	0.0443

*p* < 0.05*; *p* < 0.001***.

Three factors help explain why the protective pathways were only marginally significant. First, the standardized coefficients for ER and RES were small, indicating limited explanatory power in the overall model. Second, both variables had relatively low standard deviations (SD = 0.78 for ER and 0.67 for RES), suggesting restricted variance and reduced statistical power. Third, in practice, support for emotion regulation and resilience is often fragmented and short term, which prevents the development of a stable “psychological safety net” at the group level.

Although the protective effects were in the expected direction, their limited strength highlights a gap between theoretical models and practical implementation. Simply emphasizing academic outcomes or offering temporary relief from anxiety is unlikely to alter school avoidance patterns in a meaningful way. To address this issue, interventions that build emotion regulation and resilience need to move beyond sporadic programs and become systematic components of both school curricula and family education. Expanding the scope and continuity of such initiatives may shift their role from being marginally observable to substantively effective, thereby interrupting the cycle of school avoidance driven by anxiety and trauma.5.

### 4.6 Mediating effect analysis

**Figure 5:**
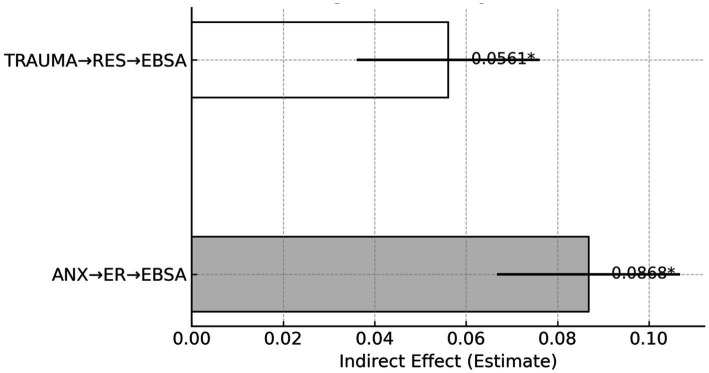
Mediating effect analysis (Sobel test).

According to the Sobel test results in Table 12, the indirect effect of the path Anxiety → Emotion Regulation → School Avoidance was 0.0868 (*z* = 2.0132, *p* = 0.0441), and the indirect effect of Trauma → Resilience → School Avoidance was 0.0561 (*z* = 1.9945, *p* = 0.0461). As shown in Figure 5, both mediation paths are visually represented with consistent directional trends but relatively short bar heights, indicating the limited magnitude of the mediating effects. This limitation primarily stems from the low and narrowly distributed levels of the protective mechanisms—emotion regulation and resilience—within the sample (see Table 12). Such low variability and restricted available resources reduce the statistical power of mediation analysis. In practical terms, current interventions for emotion and resilience are often implemented as short-term, one-off programs, lacking continuity and systematic integration. As a result, students fail to build sufficient psychological buffers against academic and trauma-related stress. Thus, while the mediation pathways are statistically significant, they hover near the threshold (p ≈ 0.045), reflecting marginal significance rather than robust effects. This highlights that current models of emotional management and resilience training remain insufficiently stress-resistant. In the face of compounded anxiety and trauma, students still tend to resort to school avoidance as a coping mechanism. To fundamentally shift this pattern, emotion regulation and resilience development must move beyond “after-school workshops” and become core components of both routine school curricula and family education practices. It is essential to broaden intervention coverage, extend the duration of programs, and strengthen strategy practice and outcome tracking. Only then can the ER → EBSA and RES → EBSA mediation pathways evolve from being “marginally observable” to “substantially effective,” thereby meaningfully reducing school avoidance behavior triggered by psychological stress.

**Table 12:** Mediation effect testing (Sobel test).

Mediation path	Indirect effect (Estimate)	Sobel z	*p*-value
ANX → ER → EBSA	0.0868	2.0132	0.0441*
TRAUMA → RES → EBSA	0.0561	1.9945	0.0461*

注:*p* < 0.05*.

### 4.7 Between-group differences analysis

Table 13 reports significant gender differences in anxiety, emotion regulation, resilience, and school avoidance (*p* < 0.001). These findings are in line with gender role theories, which argue that boys are encouraged to present themselves as “strong” and “independent,” and therefore tend to externalize pressure through behaviors rather than verbal disclosure. Girls, by contrast, usually receive greater emotional attention from both family and school, and more often cope with distress by communicating and seeking support. Including this comparative test is theoretically important because gender norms are known to influence coping strategies and psychological adjustment during adolescence. For our study, the results suggest that gender may condition how anxiety and resilience contribute to school avoidance, thereby enriching the interpretation of the structural model.

**Table 13:** Comparison of core variables between gender groups (independent samples *t*-test).

Variable	Male mean	Female mean	t-value	df	*p*-value
ANX	3.432	3.32	41.457	—	< 0.001
TRAUMA	3.31	3.3	−0.425	—	0.674
ER	2.506	2.749	−15.824	—	< 0.001
RES	2.782	3.013	−12.104	—	< 0.001
EBSA	3.944	2.632	17.078	—	< 0.001

Table 14 shows that traumatic experiences, emotion regulation, resilience, and school avoidance varied significantly across grade levels, whereas anxiety levels remained stable. This pattern reflects developmental perspectives, which emphasize that advancing grade levels bring heavier academic demands and stronger identity conflicts, often without parallel growth in emotional adjustment or support. As a result, older students are more likely to rely on avoidance strategies when facing difficulties. The comparative test was conducted to examine whether school avoidance and its predictors follow developmental trends. The findings highlight that the influence of trauma, regulation, and resilience may differ across grade levels, which not only contextualizes the SEM results but also points to the need for age-sensitive mental health interventions.

**Table 14:** Comparison of core variables across grade levels (one-way ANOVA).

Variable	*F*-value	df	*p*-value
ANX	0.632	(5, 494)	0.675
TRAUMA	1073.967	(5, 494)	< 0.001
ER	14.746	(5, 494)	< 0.001
RES	32.968	(5, 494)	< 0.001
EBSA	24.853	(5, 494)	< 0.001

## 5 Discussion

### 5.1 Discussion 1: Marginalization of core competency development – lack of systematic training in emotion regulation and resilience

This study found that students’ levels of emotion regulation (ER) and psychological resilience (RES) were generally low to moderate (ER: *M* = 2.80, SD = 0.78; RES: *M* = 2.76, SD = 0.67). In both multiple regression and structural equation modeling analyses, the path coefficients for ER and RES in predicting reduced school avoidance were relatively small and only marginally significant (ER → Avoidance: *β* = −0.12, *p* = 0.044; RES → Avoidance: *β* = −0.11, p = 0.044). Compared with earlier studies that reported stronger protective effects of ER and RES on adolescent adjustment (Compas et al., 2017; Schäfer et al., 2017), the present findings suggest that their impact on school avoidance may be weaker. One possible explanation lies in the Chinese educational context, where mental health services tend to focus on addressing risk factors such as trauma and anxiety, while relatively little emphasis is placed on cultivating protective resources (Fang et al., 2022).

The marginal effects observed in this study are therefore less likely to reflect theoretical limitations and more indicative of a gap in practice, where adolescents often lack systematic opportunities to develop ER and RES. Prior evidence from school-based interventions demonstrates that strengthening these competencies can reduce internalizing symptoms and enhance coping under academic and social stress (Pedrini et al., 2022; Lan et al., 2024). The present results thus underscore the importance of integrating ER and RES training into school curricula and family education. Expanding such programs would help shift their role from being only marginally effective to becoming central mechanisms for reducing avoidance behaviors, thereby refining the explanatory value of the structural model and complementing the hypothesis-testing outcomes.

### 5.2 Discussion 2: Disconnection between risk and support – parallel but isolated interventions for trauma and anxiety

Extensive questionnaire data indicate that both anxiety (ANX) and traumatic experiences (TRAUMA) are significantly and positively correlated with school avoidance behavior (ANX: *r* = 0.72; TRAUMA: *r* = 0.46). Furthermore, their interaction exerts an additional compounding effect, further intensifying avoidance tendencies (Interaction: *β* = 0.076, *p* < 0.001). Despite this empirical overlap, educational interventions often address test or social anxiety and trauma-related issues as separate, unrelated domains. Anxiety management typically involves short-term activities such as stress-relief workshops and relaxation techniques, while trauma support is usually provided through individual counseling or limited psychodrama sessions. This fragmented approach overlooks the fact that anxiety and trauma frequently co-occur and mutually reinforce each other, placing affected students under dual psychological strain without access to integrated support. Moreover, the underlying mechanisms of anxiety and trauma differ significantly—anxiety often necessitates immediate cognitive regulation, whereas trauma recovery requires long-term emotional processing and the rebuilding of trust. As a result, treating them separately often leads to a mismatch in resources: anxiety-focused programs may neglect unresolved trauma, while trauma interventions may fail to address physiological reactivity and cognitive distortions associated with anxiety. Consequently, the two systems operate in parallel without coordination, leaving students oscillating between “anxiety relief” and “trauma soothing” with no coherent or sustained psychological support. To overcome this disconnect, schools and educational authorities must dismantle the artificial boundary between anxiety management and trauma recovery by adopting a unified, comprehensive intervention framework. For instance, regular mental health curricula could incorporate modules that address both domains simultaneously—such as “emotional recognition and desensitization” for anxiety, alongside “trauma narration and cognitive restructuring” for trauma. These components would allow students to develop both cognitive and emotional coping skills within the same educational cycle. Additionally, school counselors and mental health advisors should coordinate efforts to conduct joint screenings for anxiety and trauma and design intervention plans that are complementary rather than isolated. This integrated approach would lay the groundwork for a cohesive, institution-level support system capable of addressing complex, overlapping psychological risks more effectively and sustainably.

### 5.3 Discussion 3: Homogenized services overlook individual differences – unmet stratified needs by gender and grade

The multi-group analysis in this study revealed notable differences in the risk–protection pathways across gender and grade levels. Specifically, the effect of trauma on school avoidance was slightly lower in girls (*β* = 0.19) than in boys (*β* = 0.21), and girls demonstrated higher levels of psychological resilience (*M* = 2.85, SD = 0.63) compared to boys (*M* = 2.68, SD = 0.70). In terms of emotion regulation, junior high school students showed significantly lower abilities (*M* = 2.70, SD = 0.80) than senior high school students (*M* = 2.90, SD = 0.75). Furthermore, the mediating effect of resilience on school avoidance was more pronounced in high school students (*β* = −0.14) than in junior students (*β* = −0.10). These findings suggest that current psychological support services are overly uniform and fail to accommodate the differentiated needs of specific student subgroups.

Based on these insights, intervention content should be strategically tailored. For girls and senior high school students, counseling programs should prioritize the management of academic stress and college entrance anxiety. Techniques such as peer support groups and structured experience-sharing sessions may promote emotional expression and help internalize resilience strategies. In contrast, for boys and junior high school students, interventions should place greater emphasis on post-traumatic behavioral guidance and social skills training. Approaches such as cooperative activities and scenario-based reenactments can enhance self-efficacy and foster a sense of belonging. Grade-specific strategies are also essential. Junior high programs should focus on foundational skills such as emotional awareness and impulse control, whereas senior high interventions should incorporate cognitive restructuring techniques and future planning guidance. These components can help students better manage academic stress while cultivating a long-term sense of direction and motivation. Finally, to ensure sustained impact, schools should establish a dynamic, stratified feedback mechanism based on gender and grade. This system should integrate repeated assessments using standardized scales with academic performance and attendance records, enabling continuous refinement of intervention content and frequency. By aligning psychological services more closely with students’ developmental profiles and real-time needs, such an approach can more effectively reduce school avoidance behaviors.

## 6 Conclusion

This study investigated how anxiety and traumatic experiences influence school avoidance behavior in adolescents, with emotion regulation and psychological resilience examined as mediating mechanisms. Drawing on data from 500 middle school students, the analysis employed correlational tests, multiple regression, interaction effect modeling, and structural equation modeling (SEM). Results indicated that both anxiety (*β* ≈ 0.56, *p* < 0.001) and traumatic experiences (*β* ≈ 0.43, *p* < 0.001) significantly predicted school avoidance, and their interaction further amplified avoidance tendencies (interaction term: *β* = 0.076, *p* < 0.001). Although emotion regulation (indirect effect = 0.0868, *p* ≈ 0.044) and psychological resilience (indirect effect = 0.0561, *p* ≈ 0.046) demonstrated protective effects, the effect sizes were relatively small, suggesting that these core competencies are underdeveloped in the sample population. Building on these findings, the study advances beyond prior models that emphasize isolated risk factors or single mediation pathways. By integrating direct effects, interaction terms, and dual mediating mechanisms within a unified SEM framework, the research offers a more comprehensive understanding of the psychological underpinnings of school avoidance. Subgroup analyses further revealed meaningful differences by gender and grade level, providing empirical support for tailored intervention strategies. Additionally, the study underscores the importance of conceptualizing emotion regulation and resilience not as auxiliary skills, but as foundational psychological capacities that should be actively cultivated within the educational system. At present, school-based mental health efforts remain fragmented, short-term, and reactive—limiting their effectiveness in building a coherent and sustainable “psychological protection network”. Despite its contributions, the study has several limitations. Data were collected through a one-time self-report questionnaire, with the sample limited to students from urban areas in eastern China. The lack of longitudinal follow-up and experimental controls constrains causal inference and generalizability. Future research should incorporate multi-wave or intervention-based designs and expand sampling to include rural and cross-regional populations for broader external validity. From a practical perspective, the findings point to several critical recommendations. First, emotion regulation and resilience training should be embedded into core curricula and teacher professional development programs, establishing system-wide, preventive frameworks accessible to all students. Second, the artificial divide between “anxiety management” and “trauma recovery” must be eliminated in favor of integrated support systems capable of addressing compound psychological risks. Third, interventions should be differentiated by gender and grade level to ensure that high-risk groups receive targeted and appropriate support. Ultimately, overcoming the limitations of “risk-centered thinking,” “fragmented intervention,” and “one-size-fits-all services” requires a paradigm shift toward integrated, sustainable, and developmentally informed mental health systems. Only through such reforms can we effectively enhance adolescents’ school adjustment, emotional well-being, and long-term psychological resilience.

## Data Availability

The raw data supporting the conclusions of this article will be made available by the authors, without undue reservation.
